# Pre-surgical Nasal Molding of a Two-Day-Old Neonate With Complete Unilateral Cleft Lip and Palate Using Passive Nasoalveolar Molding Technique: A Case Report

**DOI:** 10.7759/cureus.51822

**Published:** 2024-01-07

**Authors:** Abhijeet Jaiswal, Virat Galhotra, Saraa Angel L, Rhea Gandham

**Affiliations:** 1 Orthodontics and Dentofacial Orthopaedics, All India Institute of Medical Sciences, Raipur, IND; 2 Pediatric Dentistry, All India Institute of Medical Sciences, Raipur, IND; 3 Orthodontics and Dentofacial Orthopaedics, All India Institute of Medical Sciences, New Delhi, IND

**Keywords:** presurgical nasoalveolar moulding, presurgical infant orthopedics, passive molding, unilateral cleft lip, cleft lip & palate

## Abstract

The occurrence of congenital deformities like cleft lip and palate is not uncommon and is often a traumatizing experience for families. The entire rehabilitation process includes frequent hospital visits and the brunt of numerous procedures. Early intervention with pre-surgical infant orthopedics facilitates better surgical outcomes and additional psychosocial benefits to the infant's family. The present clinical report addresses the pre-surgical management of a non-syndromic two-day-old female baby whose parents presented with the chief complaint of deformed lips, nose, and difficulty while feeding. The neonate had a complete left-sided cleft lip, alveolus, and cleft palate on examination. Early management with pre-surgical passive nasoalveolar molding (PNAM) has favorable outcomes, including desired upper lip, alveolus, and nose shape. Non-invasive pre-surgical intervention with PNAM reduces the severity of the deformities before the primary surgical repair, thus decreasing the overall cost of cleft care and the number of secondary revisions, thus increasing the probability of favorable outcomes.

## Introduction

In 2022, Salari et al. [1] reported the global prevalence of cleft palate in every 1000 live births as 0.33, while that of cleft lip was 0.3 in every 1000 live births. The cleft lip and palate prevalence in every 1000 live births was 0.45 [1]. There is a significant unmet need for cleft lip and/or palate (CL/P) care in India [2]. The households affected by this condition have a decline in their overall quality of life and face high financial burdens. The affected patients suffer from significant impairments in speech, hearing, nutrition, and psychosocial development [3].

Throughout history, there have been attempts to reduce the surgical challenges associated with cleft lip and palate, leading to the emergence of pre-surgical infant orthopedics (PSIO) [4]. Nasoalveolar molding (NAM) is a treatment modality that aims to reduce the complexity of the defects for easier surgical repair to achieve a better outcome. The rationale behind using NAM dates back to 1984, when Matsuo et al. proposed non-surgical correction of auricular deformities, utilizing the plasticity of the infant cartilage [5]. High circulating maternal estrogen in the infant's bloodstream during the first six weeks of life raises the levels of hyaluronic acid, which in turn increases the plasticity of the tissues. Later, when the plasticity decreases after the first six months of life, the elasticity thus imparted maintains the shape of the molded tissues [4]. The NAM appliance is a removable acrylic alveolar molding plate made from a dental cast of the infant's maxilla. Embedded into the anterior portion of the molding plate is a 0.032-inch stainless steel wire, with the nasal stent bent into an "S" shape [4]. The nasal stent and the intraoral molding plate are adjusted weekly or biweekly to progressively correct the nasal and alveolar deformities, giving rise to the name nasoalveolar molding [6]. Many studies have reported a positive clinical trend using NAM without adverse effects [7,8]. Management of a neonate with a cleft lip and palate is challenging and involves a multidisciplinary approach. This case report demonstrates the management of a two-day-old baby with a complete unilateral cleft lip, alveolus, and palate using passive NAM.

## Case presentation

Case description

The parents of Indian descent were referred from the Pediatric Department to the Department of Dentistry at the All India Institute of Medical Sciences (AIIMS), Raipur, Chhattisgarh, with their non-syndromic two-day-old female baby, with a chief complaint of malformed lips, nose, and difficulty in feeding due to regurgitation of milk. Detailed case history revealed a non-consanguineous marriage with an insignificant family history of cleft lip and/or cleft palate on the maternal and paternal sides, with no other hereditary disorders. The neonate's birth history was uneventful, with an optimal birth weight of 2.84 kg and no other systemic illness or past intervention.

Clinical findings

**Figure 1:**
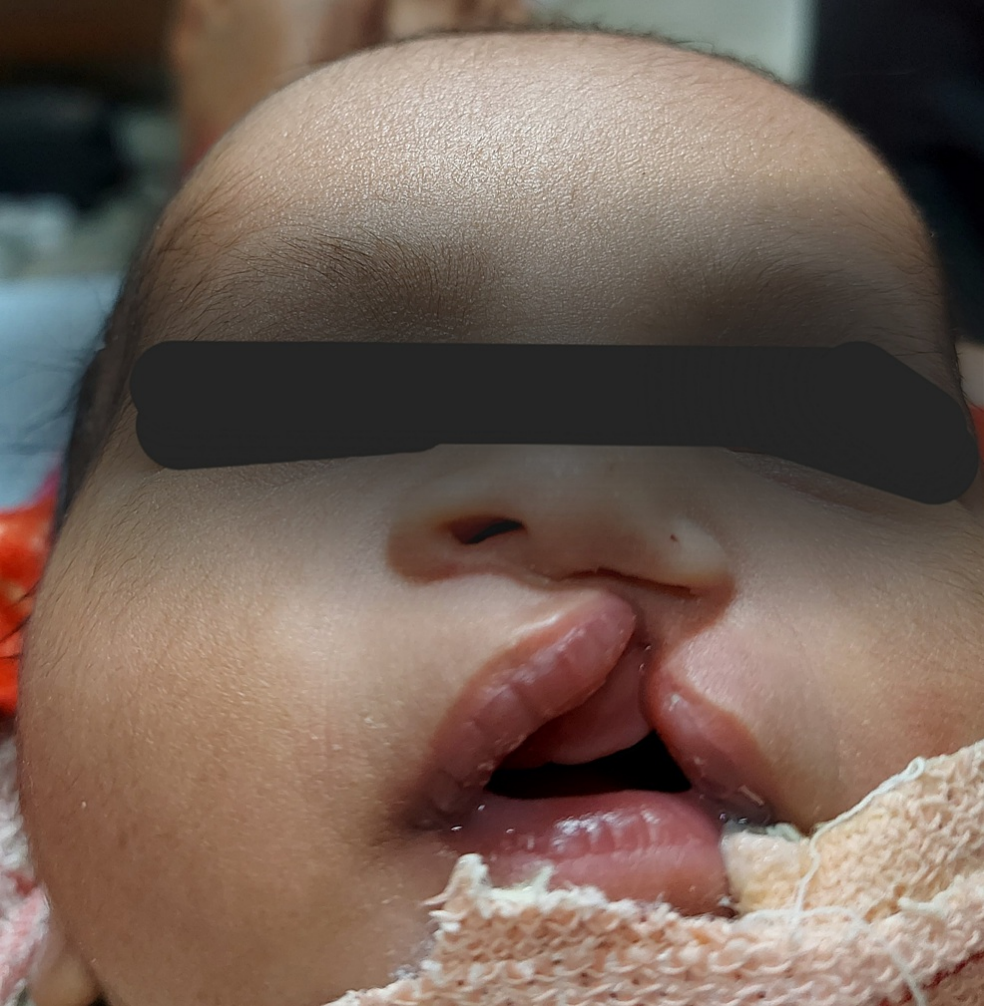
Pre-treatment unilateral cleft lip and palate with asymmetric upper lip segments

On examination, the moderately built two-day-old neonate had a complete left-sided cleft lip, alveolus, and palate, asymmetric nostrils, and a depressed ala of the nose on the affected side. The columellar height was reduced, and the nasal septum was deviated to the normal side. The intraoral examination revealed a satisfactory alignment of the major and minor segments with no overlapping and an alveolar cleft of 4 mm (Figure 1).

**Figure 2:**
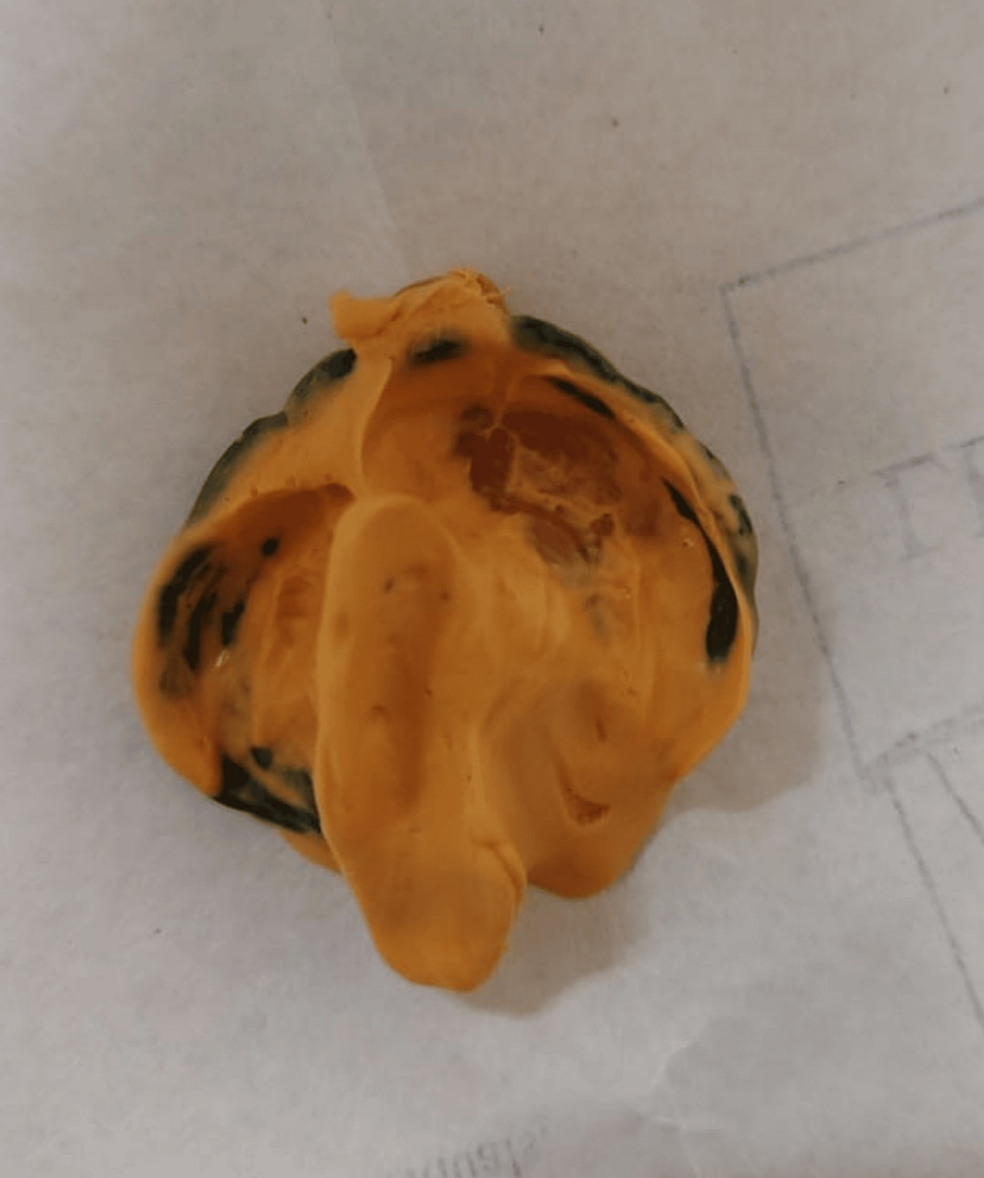
Final impression

Considering the patient's age and alignment of the maxillary segments, there was no requirement for alveolar molding and hence the pre-surgical nasal molding incorporated in a passive NAM plate was planned before lip repair, followed by cleft palate repair. The treatment procedure was elucidated to the parents during the initial appointment, and their agreement was acquired. Per department protocol, an early morning visit was arranged for impression-making in the presence of an anesthetist and oral surgeon. The procedure was explained to the mother, and an impression was made with the neonate in the prone position in the mother's lap with her head slightly downwards. The initial impression was taken using the fast-setting putty material, and a customized tray was fabricated. The final impression was made using the custom tray with a light-body silicone material after border molding. A 25-40 micron thick cellophane acted as the barrier film on top during impression making to ensure complete material retrieval without entering the undercuts (Figure 2).

**Figure 3:**
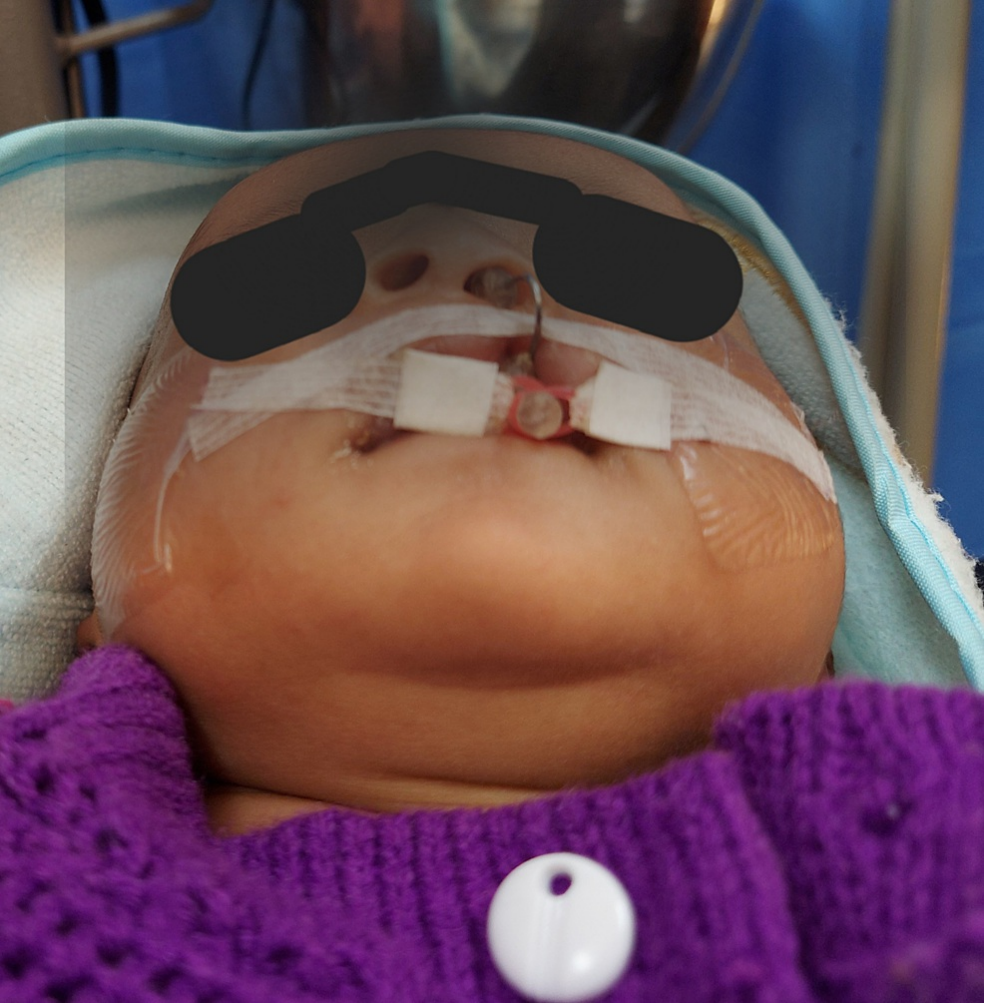
Pre-surgical nasal molding with passive nasoalveolar molding (in place) and lip taping

High-volume evacuation was used to drain out fluids. The baby was encouraged to cry to ensure airway patency. A customized passive NAM plate covering the entire palate and alveolar ridge was fabricated after blocking the cleft with wax and delivered immediately. The patient's parents were asked to report immediately in case of any signs of discomfort. Infant suckling with the plate was evaluated after two days, and parents were educated regarding the feeding. The lip taping was initiated on the fifth day of birth as per Grayson protocol [4]. A "swan neck"-shaped nasal stent made of 0.032-inch titanium molybdenum alloy (TMA) wire was added on the eighth day, exiting from the labial portion of the molding plate and contacting the medial side of the septal cartilage to lift the alar dome. The nasal end of the stent was covered with a bilobed soft resin capping with the upper lobe contacting the nasal mucosa (Figure 3).

**Figure 4:**
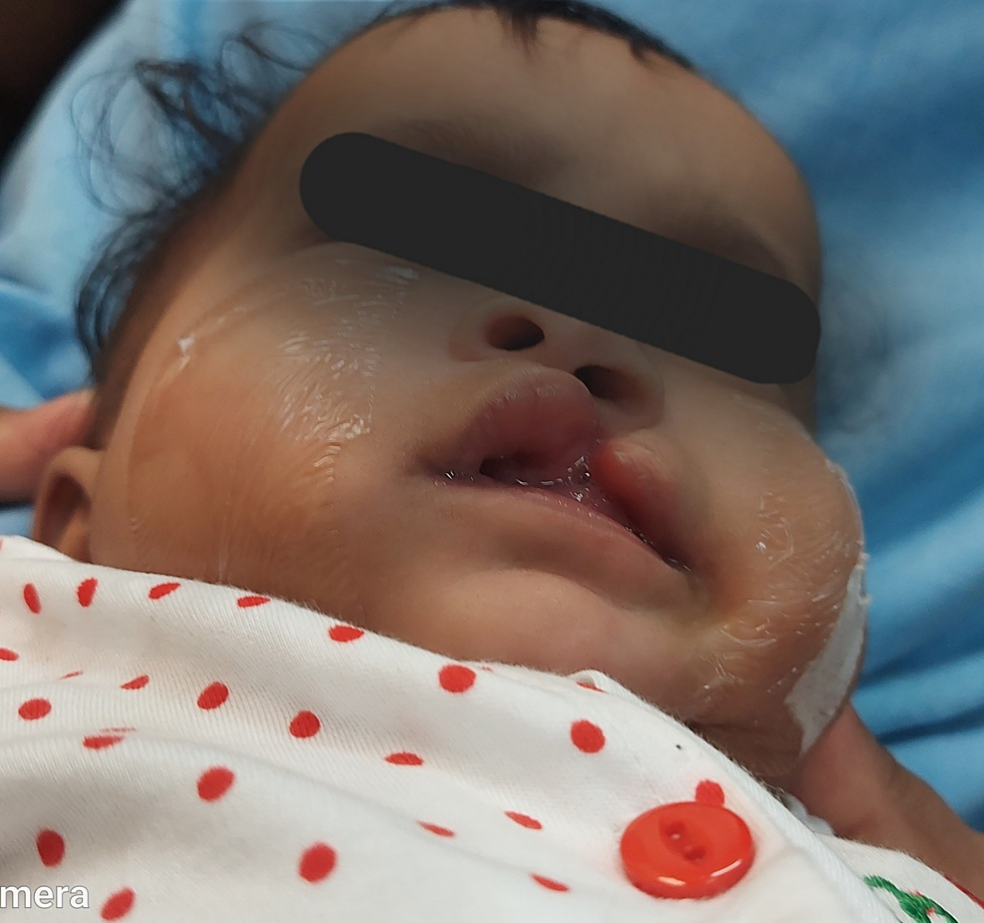
Immediate post-operative stage after seven weeks of nasal molding

The stent was activated biweekly until slight blanching was seen by alternative wire activation and the addition of the soft resin in the upper lobe. Periodic adjustment and activation of the stent were done along with continued lip taping to improve nasal and lip symmetry with regular follow-up after rectifying pressure marks or mucosal irritation. After seven weeks, the narrowing of the interlabial cleft segment defect and improvement in the alignment of lips were observed, along with increased philtrum length, and increased columellar height almost equal to the normal side (Figure 4).

There was an improvement in the patient's acceptance of plate and feeding, as reported by the parents. At two and a half months, the patient underwent primary cleft lip repair through the modified Millard technique in the Department of Oral Surgery, AIIMS, Raipur. The patient was followed up till the palate surgery was performed at one and a half years of age and achieved a satisfactory esthetic outcome.

## Discussion

Infants born with oral clefts are generally malnourished and anemic, with low birth weight that compromises their early surgical intervention [8]. Nasal and alveolar molding techniques have been considered to positively affect lip, nasal, and alveolar surgical repairs by reducing muscle tension and successfully decreasing defects in short-term evaluation [9]. In addition to the approximation of the segments' early intervention of the cleft defect, the molding plate has many advantages, including nutritional gain, preventing nasal regurgitation, the establishment of normal sucking movement, and restoring the tongue to a downward position, thereby preventing its protrusion into the defect [10]. Although feeding plates have been delivered within a few hours of birth up to the initial weeks of the neonatal period, there are no reports of early nasal molding starting within a week of birth [11,12]. Nasal molding is usually initiated after the alveolar molding when the cleft segments are approximately at least 5 mm [13]. In this case, the authors planned a passive NAM plate over the classical pre-surgical nasoalveolar molding (PNAM), as the initial alveolar cleft repair was favorable. Moreover, the authors hypothesized that the early nasal molding would result in a more stable outcome with less relapse.

**Figure 5:**
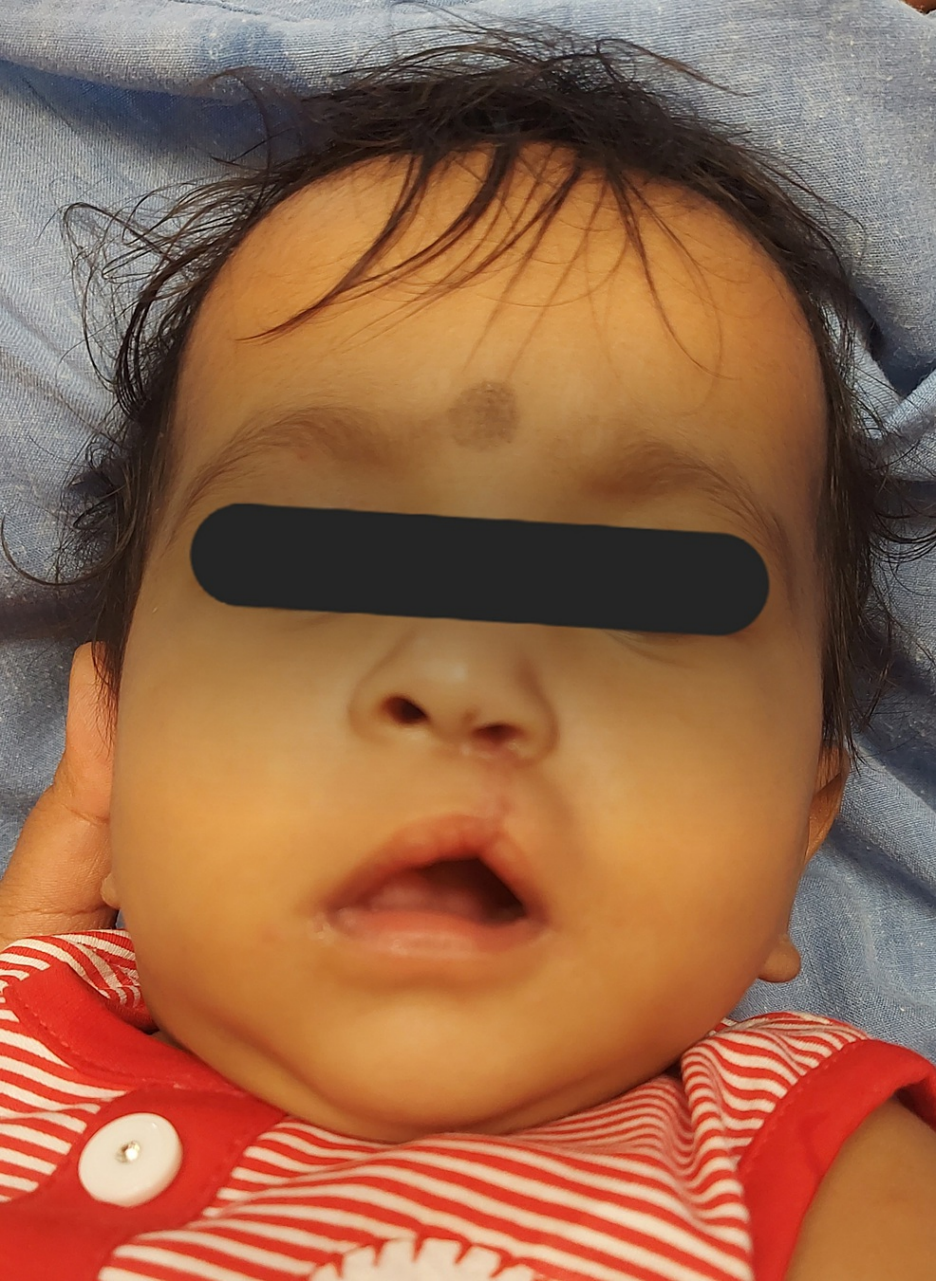
Post-treatment follow-up at six months after cleft lip repair

The lip taping approximates the base of the nose while the nasal stent pulls the columella upwards and laterally. Taping causes the tissue creep of the lips over time. It aids in approximation with less tension, causing passive molding of the underlying alveolus, as explained by Hotz, and generating simultaneous lengthening of the philtrum and columellar height and width due to traction [14]. Recent randomized control trials have concluded that lip taping alone can reduce cleft defects like active PNAM, indicating the pivotal role of nasal molding [15]. Nasal molding repositions the deviated nasal tip, especially in bilateral cleft lip and palate cases with the neighboring tissues helping to center the extremely malpositioned premaxilla [16]. Hence, nasal molding using springs and external acrylic stents has also been successfully used [17,18]. Punga et al. observed that patients with nasal stents incorporated into the PNAM plate showed a significant increase in columellar height and nasal tip projection compared to those treated with just the PNAM plate [19]. Pai et al. assessed the alveolar cleft width and nostril symmetry after PNAM therapy. It showed the height, columella angle, width, and relapse of 20%, 4.7%, and 10% at one year of age [20]. In this clinical report, the authors have also noted significant improvement in columellar height, width, and tip centering that was stable six months after the cleft lip repair. A slight yet significant relapse at one-year follow-up was noted due to differential growth of the cleft and non-cleft side. Nevertheless, early intervention still resulted in stable results over time than the delayed start of nasal molding (Figure 5).

Difficulties in early treatment of the NAM include parental cooperation, systemic health of the patient, repeated airway infections, and local inflammatory causes [10]. Therefore, under favorable conditions, nasal molding can be started within a week of birth.

## Conclusions

Passive NAM with nasal stent is an excellent adjunct in the early management of cleft anomalies in neonates with minimal alveolar cleft and favorable alveolar arch alignment. It provides a safe, effective, and lasting improvement in the esthetics of the nasolabial complex in infants with unilateral and bilateral cleft deformities, reducing the burden of care. It, however, requires active participation and compliance from the patient's caregivers.

Patient perspective

Highly satisfied and grateful for the treatment provided. Adequate restoration of esthetics and function eased the psychosocial burden and significantly improved the quality of life, thus making it a life-changing experience.
